# Hospital, health, and community burden after oil refinery fires, Richmond, California 2007 and 2012

**DOI:** 10.1186/s12940-019-0484-4

**Published:** 2019-05-16

**Authors:** Linda L. Remy, Ted Clay, Vera Byers, Paul E. Rosenfeld

**Affiliations:** 1 Family Health Outcomes Project, Family and Community Medicine, School of Medicine, University of California San Francisco, 500 Parnassus Ave. Room MU-337, San Francisco, CA 94143-0900 USA; 2 Immunology Inc, PO Box 4703, Incline Village, NV 89450 USA; 3 SWAPE, 2656 29th Street, Suite 201, Santa Monica, California 90405 USA

**Keywords:** Chemical release incident, Emergency department, Emergency medical services, Oil refinery, Particulate matter, Small area analysis, Natural experiment, Environmental epidemiology

## Abstract

**Introduction:**

Emergency Departments experience a significant census burst after disasters. The aim of this study is to describe patient presentations at Emergency Departments in Contra Costa County, California following chemical release incidents at an oil refinery in 2007 and 2012. Specific areas of focus include hospital and community burden with an emphasis on disease classes.

**Methods:**

Searching 4 weeks before through 4 weeks after each event, Emergency Department abstracts identified patients living in Contra Costa County and seeking care there or in neighboring Alameda County. City and ZIP-code of residence established proximity to the refinery. This provided the following contrast groups: Event (2007, 2012), time (before, after), location (bayside, rest of county), and within bayside, warned or not warned to shelter in place. Using the Multi-Level Clinical Classification Software, we classified primary health groups recorded 4 weeks before and after the events, then summarized the data, calculated rates, and made tables, graphs, and maps to highlight findings.

**Results:**

Number of visits meeting selection criteria totalled 105020 records. Visits increased modestly but statistically significantly after the 2007 incident. After the 2012 incident, two Emergency Departments took the brunt of the surge. Censuses increased from less than 600 a week each to respectively 5719 and 3072 the first week, with the greatest number 2 days post-event. It took 4 weeks for censuses to return to normal. The most common diagnosis groups that spiked were nervous/sensory, respiratory, circulatory, and injury. Bayside communities had statistically significant increases in residents seeking care. Specifically, visits of residents in warned communities nearest the refinery increased by a factor of 3.7 while visits of residents in other nearby un-warned communities increased by a factor of 1.5.

**Conclusions:**

The 2012 Emergency Department census peaked in the first week and did not return to normal for 4 weeks. Diagnoses changed to reflect conditions associated with reactions to chemical exposures. Surrounding communities and nearby hospitals experienced significant emergent burdens. In addition to changes from such events in patient diagnoses and community burden, the discussion highlights the long-term implications of failures to require adequate monitoring and warning systems and failures of health planning.

**Electronic supplementary material:**

The online version of this article (10.1186/s12940-019-0484-4) contains supplementary material, which is available to authorized users.

## Background

### Chemical release incidents

Some events particularly stress the emergency medical services system and require executing disaster protocols and plans. This paper compares the impact of two chemical release incidents (CRI) in Richmond, California on Emergency Department (ED) utilization, population health, and community burden in 2007 and 2012.

About 5:30 PM on Monday, 06-Aug-2012, as workers attempted to repair a hydrocarbon leak, a major fire erupted at the Chevron Refinery (Refinery) in Richmond. The Refinery is located in western Contra Costa County (County) on the shores of San Francisco Bay. Five emergency responders had minor burns and received first aid [1]. Refinery CRI can result in fires, as here, or be a consequence of fires or other disasters.

As it had done after previous CRI at this Refinery, the County activated a Level 3 Community Warning System notification for Richmond, North Richmond, and San Pablo, with phone calls and sirens warning people to go indoors and shelter-in-place (SIP) [2]. Residents of warned communities live within 4–5 miles of the Refinery. Some calls notifying residents to SIP did not occur until over 4 hours after the release [3]. The County initiated the Multi-Casualty Incident Plan at “Tier 0”, denoting a major incident with no reported casualties, with alerts to hospitals, ambulance services, and other key health service personnel.

Nearby EDs were quickly overwhelmed and put on ambulance diversion. The 911 system also was overwhelmed, with ambulances responding to nearby communities and with EDs outside the immediate area experiencing surges. Due to the large number of patients seeking emergency care and calling ambulances, the County upgraded the Multi-Casualty Incident Plan to Tier 3, incident with mass casualties or potential for mass casualties [2]. Most people did not require inpatient care. However, the number of patients seeking treatment continued to surge for weeks.

The Refinery has a history of less serious CRI requiring SIP warnings: Monday 12-Apr-1989 [4], Thursday 25-Mar-1999, Thursday 31-Jan-2002, Wednesday 16-Sep-2003, and Monday 15-Jan-2007 [1]. This study focuses on the 2007 and 2012 CRI.

The County is home to other major refineries with their CRI histories before and after 2012 [1]. Two are in nearby Rodeo and two are to the immediate east of Rodeo in Martinez along the County northwest shore, in turn about 10 and 20 miles from the subject Refinery. One Rodeo refinery paid nearly a million dollars in fines since 2014 [5] and currently is seeking permits to become a major tar sands processor [6, 7]. These refineries and all towns affected by the 2012 CRI are 0–10 miles from the Hayward Fault upwelling [8, 9]. Earthquakes along the San Andreas Fault to the west and the Hayward Fault formed San Francisco Bay.

### Health impact of chemical release incidents

Human health can be affected by exposure to various types of particle pollution from major industrial or natural CRI as well as by chronic exposure to power plant by-products [10, 11]. Events such as Seveso, Three Mile Island, Bhopal, Chernobyl, World Trade Center, and Fukushima [12] are examples of major CRI. Earthquakes [13–15], train wrecks [16, 17], hurricanes [18, 19], and wildfires [20, 21] also include CRI. Environmental contextual factors such as the nature of the incident (natural, technological, unintended, or deliberate), duration, setting, and size influence the ability of responders to care for victims [22]. A systematic review of seven decades of disaster management literature found that less than 1 in 5 of 9433 articles used a quantitative methodology to understand these events and concluded that an evidence-informed approach to disaster management is needed [23].

Smoke from chemical releases and fires contains various carcinogens and un-combusted hydrocarbons with particulate matter (PM) of various sizes. The most dangerous, called “ultrafine”, are less than 2.5 uM (micrometer). This PM size can deposit in the trachea and bronchial tree and can be inhaled as deeply as the alveoli [24].

The first acute effects of inhalation are irritation to the eyes, nose, and throat. Toxicities are principally respiratory, for example, acute asthma in children [25, 26]. Respiratory effects are rapid and occur with only a one-hour exposure [27], with the lungs usually bearing the most serious impact. The cardiovascular system is also involved, either secondary to pulmonary damage or directly [28]. For example, during the brief time cleaning up after a fire without breathing protection, firefighters have a significant decrease in pulmonary function with evidence of increased alveolar capillary membrane permeability [29].

Smoke inhalation produces long term pathology that includes worsening of asthma and cardiovascular effects such as stroke and cardiac disease [21, 30, 31]. People with existing heart or lung disease, people with diabetes, older adults, children, and people of lower socio-economic status have greater risk of particle pollution health effects [8, 9, 32].

Chronically exposed to PM from various refineries ringing its shores and crosshatched with several highly-trafficked freeways, County residents of all age groups have higher rates of asthma-related ED visits, hospitalizations, and deaths as compared to Californians state-wide [33], with west County residents having extremely high rates [34]. The west County region has six times more diesel air pollution per square mile annually (more than 5 tons) than the County as a whole (0.8 tons), and 40 times more than California state-wide (0.1 ton) [35].

### Importance of adequate air monitoring

In a large industrial fire, smoke and its associated chemicals may affect residents in both nearby and upwind or downwind neighborhoods. Unfortunately, no air monitoring was done for the 2007 event, and no reliable air quality data exists for the 2012 event. Monitoring stations were not located near affected neighborhoods, and monitoring did not begin until after emergency responders extinguished the fire [36].

Fence-line, community, and mobile monitoring are standard air monitoring methods, and each serves a distinct purpose. Fence-line monitoring primarily identifies non-routine emissions during normal operation [37]. Community monitoring provides data to develop spatial gradients of chronic exposure [38]. Mobile monitoring supplements on-going monitoring during incidents [39]. Time resolution for each method will differ, with fence-line on the order of hours, and mobile only under special circumstances.

The first monitoring after the 2012 fire was by the Bay Area Air Quality Management District’s (BAAQMD) permanent monitors, which would be classed as community monitors. Of the 40-monitor network, only five were in the western County where all refineries are located. BAAQMD monitors sample for hydrogen sulphide, elemental carbon, ozone, sulfur dioxide, and PM 10. The only reliable PM monitor, 2 miles east of the Refinery, did not sample until after the fire ended. About 2 hours after the fire ended, BAAQMD also used ambient air canisters at eight individual locations, analysing 23 compounds including benzene, toluene, and ethanol. Again, no BAAQMD monitoring assessed PM or un-combusted hydrocarbons.

The evening of the 2012 CRI, the Refinery took 17 direct-reading samples for hydrogen sulphide, sulfur dioxide, and carbon monoxide in Richmond, and collected 19 Tedlar bag samples in downwind communities to test for hydrocarbon and sulfur compounds. Over the next 2 days, the Refinery did more follow-up monitoring for hydrogen sulphide, sulfur dioxide, and carbon monoxide. However, as here, refinery fires typically are relatively short events, and sampling after the event is not helpful to show community impacts. No sampling tested for heavier hydrocarbons that could indicate the severity of pollution from a refinery fire.

If available, two other methods can measure exposure: analysis of photos of the event and meteorological analysis. Available photos show fire and smoke from four distinct locations within the Refinery boundaries, with thick, black particulate matter spreading across a large geographic area and into the atmosphere. Exact times and locations for photos were not standard, and the fire burned well past sunset. Thus, it was not possible to conclude anything but that dense black smoke was consistent with PM presence. Meteorological analysis was inconsistent because of placement of weather stations, two on a pier about 2 km southwest of the fire separated by a 300 m high ridge, and another about 2 km northeast of the fire. Such facilities may experience very different wind patterns [33].

## Materials and methods

### Overall design

To assess the impact of the Refinery CRI on ED utilization and population health, we used confidential ED data from the California Office of Statewide Health Planning and Development (OSHPD) [40] to compare visit rates 4 weeks before and 4 weeks after the 2007 and 2012 Refinery CRI by place of residence in the County. For both events, the County issued SIP warnings for the same communities. The numerator of each rate is a count of records within various categories, and the denominator is total population. Secondary analysis involved graphing count data and mapping rates.

### Hospital data

We began this study using confidential ED and Patient Discharge (PD) data previously prepared for longitudinal research [41]. These files contain medical summaries of all patient visits to EDs or admissions to hospitals licensed by the State of California. Patients admitted to inpatient care through the ED are reported in the PD files.

ED data are not available before 2005, so we focused on the 15-Jan-2007 and 06-Aug-2012 events. Following up on the Wettstein et al. methodology [18], we explored using both ED and PD data. Unlike Wettstein, studying the health impact of forest fires, we found no PD surge overall or for inpatient admissions through the ED. Given this, we dropped PD data.

Variables used include patient’s birthdate, sex, ZIP-code and county of residence, ED visit date, and principal diagnosis (PDX) classified based on the International Classification of Diseases, 9th Revision, Clinical Modification. Preliminarily selected records met the following basic criteria: sex, birthdate, and PDX present, resident of the County and seeking care in the County or neighboring Alameda County in the period 4 weeks before through 4 weeks after the 2007 or 2012 CRI. These criteria found 105020 ED visits.

To classify conditions, we used Level 1 (Body System, DXCH) and Level 2 (Disease Condition, DXCL) of the Multi-Level Clinical Classification Software (CCS) developed by the federal Agency for Healthcare Research and Quality (AHRQ) [42]. Affected body systems (DXCH) and conditions under body systems (DXCL) were identified by searching the PDX field. To identify conditions contributing to high frequency DXCH, we selected DXCL diagnosed for 500 or more patients from the BAY region in the 8-week period around the 2012 event. See Additional file 1 for descriptions of groupers to select records and classify conditions.

### Contrast groups

This work uses four contrast groups based on events, time, place, and warning. Selected events occurred in 2007 and 2012 in Richmond, California. Both events occurred on a Monday. We calculated the study frame as the period 4 weeks before and 4 weeks after the day of each event, with event day and week assigned the value zero (0). This created a 56-day interval to assess ED utilization with two 4-week windows of equal length before (days − 28- to − 1, weeks − 4 to − 1) and after (days 0–27, weeks 0 to 3) the 2007 and 2012 events. We also made a categorical time variable with two groups: before and after.

The Refinery and affected cities are geographically at the County’s western boundary along the shore of San Francisco Bay (BAY, West County) in northern California. A low mountain ridge to the east separates the primary study area BAY from the rest of county (ROC). This ridge is the upwelling of the Hayward Fault [8]. We used ZIP-code (ZIP) of patient residence to assign city, assigned cities to BAY or ROC, and within BAY, flagged ZIPS warned to SIP.

Two BAY communities received SIP warnings: Richmond (which includes North Richmond, ZIPs 94801, 94804, 94805) and San Pablo (94806). Most residents in these ZIPs live less than 4 miles from the Refinery. Other BAY communities, about 4 to 10 miles from the Refinery, did not receive SIP warnings: Rodeo (94572), Hercules (94547), Pinole (94564), El Sobrante (94803), and El Cerrito (94530). This created comparison groups based on events (2007, 2012), time (before, after), place (BAY, ROC), and within BAY, SIP warning (warned/not warned).

### Population denominator

We maintain a set of longitudinal (1970 forward) files with California population estimates by sex, age, and race/ethnicity, with ZIP-code level estimates from 1989 forward [43]. For this study, we extracted the 2007 and 2012 County ZIP-level estimates, and assigned ZIPs to BAY or ROC and warned or not warned, then summarized the data to obtain total population.

### Statistical tests

We summarized the diagnosis-classified file by 2-level event (2007, 2012), place (ZIP, BAY, ROC), time (before, after), and SIP warning status (warned, not warned), then merged the summary with the population denominator file. To calculate rates per 1000 (1 K) population, the numerator was the count of ED visits within the various categories. The denominator was the population estimate for the geographical area in the given year. To calculate 95% confidence intervals (CI) for rates, we used the Wilson Score method with continuity correction [44].

To describe difference between rates, we calculated the relative risk (RR) or risk ratio, selecting the usually higher rate divided by the usually lower rate, yielding RR values usually greater than 1.0. In the context of this study, the usually higher rates were After-CRI versus Before-CRI, BAY versus ROC, and warned ZIPs nearest the Refinery versus not warned. Upper (UCL) and lower (LCL) confidence limits for the RR were calculated using the method described for SAS® Proc Freq, “Relative Risk Estimates”, “Cohort Studies” [45]. Two-tailed *p*-values corresponding to the CL were obtained by setting the formula for the LCL equal to 1.0, solving for the Z-score, and obtaining the corresponding probability from the normal distribution.

The term “significant” used in this study combines both statistical and clinical criteria, specifically a LCL of 1.05 or more, and p-value of .01 or less. The purpose is to minimize trivial findings [46, 47]. In tables, we show decimals as needed to document CL width. In addition, we bold column RR values that are 1.10 or more and meet the above criterion for “significant”. In the text, we report significant rates without the CL. We also developed graphs to show the changing surge by time, grouping cities by whether residents were or were not told to SIP.

All programming was in SAS 9.4. Programs to prepare data and calculate statistics used SAS macros developed by our group. SAS formats [48] and macros [49] are available on our website. Programs specific to the work reported here are available on request. The map was made using ArcMap 10.6. Human subjects protocol restrictions do not allow data sharing. However, researchers with the same files and our programs would be able to replicate our results.

## Results

Table 1 shows demographic characteristics for the County and the census of ED visits by area and event year. The County reflects the diverse population of the larger San Francisco Bay Area. With a County population of about 1 million, about 27% live in West County (BAY).

**Table 1:** Demographic characteristics of County population and ED census by area and year

Source	Characteristic		Group	BAY		ROC	
				2007	2012	2007	2012
Population			Total	213145	223021	784512	816780
	Age	Number	0 to 14	45042	42940	169791	160691
			15 to 44	91049	92986	308188	311279
			45 to 64	53193	59865	214998	232419
			65 up	23861	27230	91535	112391
		Percent Total	0 to 14	21.1	19.3	21.6	19.7
			15 to 44	42.7	41.7	39.3	38.1
			45 to 64	25.0	26.8	27.4	28.5
			65 up	11.2	12.2	11.7	13.8
	Race/ Ethnicity	Number	White	57908	53198	473402	451620
			Black	47600	46406	50233	55970
			Hispanic	69727	82542	152844	180585
			API	37220	40294	105331	125973
			AIAN	689	582	2702	2631
		Percent Total	White	27.2	23.9	60.3	55.3
			Black	22.3	20.8	6.4	6.9
			Hispanic	32.7	37.0	19.5	22.1
			API	17.5	18.1	13.4	15.4
Emergency Department			Total	11658	26655	29827	36880
	Age	Number	0 to 14	2645	5605	6508	5834
			15 to 44	5053	11603	12549	15362
			45 to 64	2689	6926	6361	9657
			65 up	1271	2521	4409	6027
		Percent Total	0 to 14	22.7	21.0	21.8	15.8
			15 to 44	43.3	43.5	42.1	41.7
			45 to 64	23.1	26.0	21.3	26.2
			65 up	10.9	9.5	14.8	16.3
	Race/ Ethnicity	Number	White	4368	7918	19101	22338
			Black	4005	7673	3442	5400
			Hispanic	2579	9133	5966	6764
			API	688	1887	1269	2288
			AIAN	18	44	49	90
		Percent Total	White	37.5	29.7	64.0	60.6
			Black	34.4	28.8	11.5	14.6
			Hispanic	22.1	34.3	20.0	18.3
			API	5.9	7.1	4.3	6.2

ED caseloads are unpredictable and, by law, ED may not choose patients they accept or limit the number of patients seen. With modest changes in countywide total population, BAY had an absolute increase of 14997 ED visits (128.6%) in 2012 compared with an absolute increase of 7053 for ROC (23.6%), with huge increases in all age groups in BAY relative to ROC. For example, with about one-quarter of the County population, BAY visits for age 15–44 increased by 6550 (129.6%) in 2012 versus an increase of 2813 (22.4%) for ROC.

**Fig. 1:**
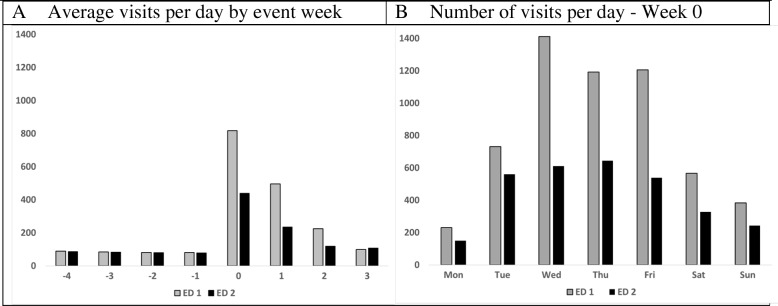
Bay Emergency Department visits during the 2012 study period

This provides a longitudinal overview of ED census changes, but does not give insight into how census changed with CRI. Figure 1a shows the average number of visits over the 8-week study period for the two EDs taking most of the burst from the 2012 CRI. Before the CRI, each ED had about 80 visits daily. The week of the CRI, average visits per day rose respectively to 817 and 439. It took about 4 weeks to return to pre-CRI levels. The 2012 CRI occurred at 5:30 PM on Monday Day 0. By midnight, when the census day changes and 6.5 h after the fire began, Fig. 1b indicates the Monday census more than doubled in ED1 (*N* = 231) and increased about 70% in ED2 (*N* = 149). In Week 0, both EDs surged for several days, with census still above average at end of week and from Fig. 1a, high through the next several weeks.

Table 2 identifies major body systems with 1000 or more cases and with number of cases at least 10% greater after than before the CRI. Comparing before and after, the table shows the number of patients with the diagnosis, rates adjusted per 1 K population with associated LCL and UCL, and symbols indicating the type of statistically significant comparison. Consistent with reports summarized earlier that identified the BAY as having chronic severe air quality problems and more illness, here we see that BAY had higher illness rates than ROC for most identified body systems before the CRI in both 2007 and 2012.

**Table 2:** Major body system rates per 1 K population by event, area, and time

Body	Event	Area	Before CRI					After CRI				
System			Visits	Rate	LCL	UCL		Visits	Rate	LCL	UCL	
Nervous Sensory	2007	ROC	1304	1.7	1.6	1.8		1449	1.8	1.8	1.9	
		BAY	452	2.1	1.9	2.3	^b^	504	2.4	2.2	2.6	^b^
	2012	ROC	1643	2.0	1.9	2.1		1760	2.2	2.1	2.3	
		BAY	583	2.6	2.4	2.8	^b^	4989	22.4	21.8	23.0	^b, c^
Circulatory	2007	ROC	882	1.1	1.1	1.2		951	1.2	1.1	1.3	
		BAY	322	1.5	1.4	1.7	^b^	321	1.5	1.3	1.7	^b^
	2012	ROC	1320	1.6	1.5	1.7		1376	1.7	1.6	1.8	
		BAY	393	1.8	1.6	1.9		827	3.7	3.5	4.0	^b, c^
Respiratory	2007	ROC	1919	2.4	2.3	2.6		2631	3.4	3.2	3.5	^a^
		BAY	878	4.1	3.9	4.4	^b^	1253	5.9	5.6	6.2	^b, c^
	2012	ROC	1340	1.6	1.6	1.7		1637	2.0	1.9	2.1	^a^
		BAY	590	2.6	2.4	2.9	^b^	4352	19.5	18.9	20.1	^b, c^
Injury/ Poisoning	2007	ROC	2748	3.5	3.4	3.6		2759	3.5	3.4	3.7	
		BAY	1063	5.0	4.7	5.3	^b^	1003	4.7	4.4	5.0	^b, c^
	2012	ROC	4161	5.1	4.9	5.3		4383	5.4	5.2	5.5	
		BAY	1268	5.7	5.4	6.0	^b^	4447	19.9	19.4	20.5	^b, c^

^a^ROC after CRI significantly higher than ROC before CRI

^b^BAY significantly higher than ROC

^c^BAY after CRI significantly higher than Bay before CRI

Nervous system/sensory system conditions that increased significantly post-CRI were migraine headaches (RR = 4.3), eye infections (RR = 6.3), other eye conditions (RR = 11.1), and dizziness (RR = 1.7). Within the circulatory system, chest pain (RR = 1.5) was the only diagnosis group with enough cases that increased post-CRI. Respiratory conditions showing an increase were asthma (RR = 2.9), upper respiratory infections (laryngitis, pharyngitis, sinusitis) (RR = 2.2), and other upper (RR = 6.8) and lower (RR = 4.5) respiratory conditions. Under injury/poisoning, non-medical poisoning (RR = 51.9) was the common factor. See Additional file 2 for details.

**Fig. 2:**
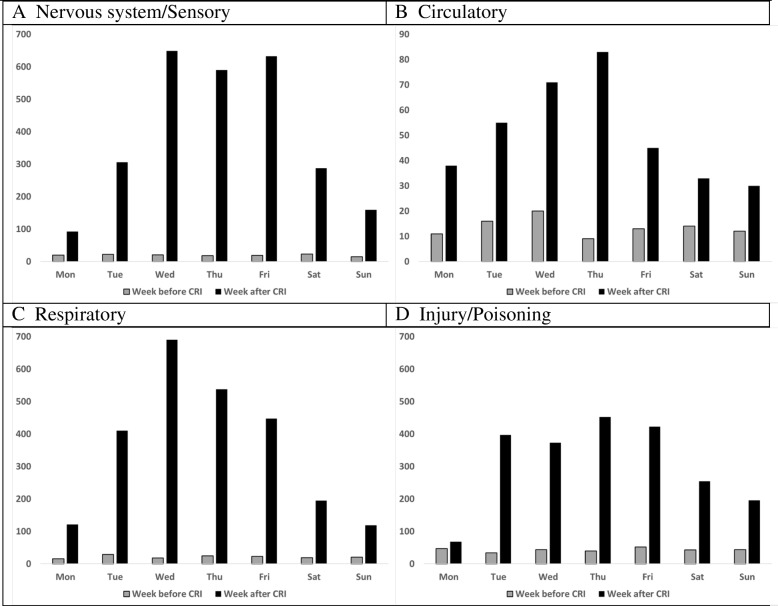
Patients (N) with affected body systems by day, week before and after 2012 CRI

Figure 2 examines changes in the number of patients by affected body system, comparing week days Monday through Sunday the week before and after the 2012 CRI. After the event, the number of patients with given diagnoses exploded. For example, consider circulatory conditions (Fig. 2b), in this case primarily chest pain. On Monday (Day 0), number of patients with circulatory conditions increased 4-fold from 11 (the previous Monday) to 43 and peaked at 83 on Thursday, almost a 10-fold increase over the previous Thursday. With post-CRI increases in the high 600 s, nervous system/sensory and respiratory systems were most affected. We also see that different conditions peak at different times following a CRI like this.

Now we turn to the community burden of these CRI. Table 3 shows -- by year, warning status, and location -- the number of ED visits in the 4 weeks before and after each CRI. The Refinery is in ZIP 94801. Overall, the 2007 CRI had a significant but modest impact near the Refinery (primarily ZIPS 94804 and 94806) (RR = 1.12), over the rest of the BAY region (RR = 1.12), and also in ROC (1.11). Among the warned zips, 94801 (where the Refinery is located), and 94805 showed no increase in ED visits (RR = 1.02 and 0.98) in spite of being warned to SIP. The 2012 CRI significantly affected all BAY communities, but not ROC.

**Table 3:** Visits by event year, warning status, and city

Year	Warned	ZIP-code	City	Visits 4-weeks		Rel Risk			
				Before	After	RR	LCL	UCL	P-Val
2007	Yes	Warned BAY (0–4 mi)		3995	4463	**1.12**	1.07	1.17	< 0.0001
		94801	Richmond	1556	1587	1.02	0.95	1.09	0.5701
		94804	Richmond	912	1124	**1.23**	1.13	1.34	< 0.0001
		94805	Richmond	247	241	0.98	0.82	1.16	0.7850
		94806	San Pablo	1280	1511	**1.18**	1.10	1.27	< 0.0001
	No	Not warned BAY (4–10 mi)		1510	1690	**1.12**	1.05	1.20	0.0006
		94530	El Cerrito	302	320	1.06	0.91	1.24	0.4501
		94803	El Sobrante	456	500	1.10	0.97	1.24	0.1299
		94564	Pinole	277	325	1.17	1.00	1.37	0.0314
		94547	Hercules	311	345	1.11	0.95	1.29	0.1554
		94572	Rodeo	164	200	1.22	1.00	1.49	0.0344
		Rest of County (10+ mi)		14127	15700	**1.11**	1.09	1.14	< 0.0001
2012	Yes	Warned BAY (0–4 mi)		4690	17312	**3.69**	3.57	3.81	< 0.0001
		94801	Richmond	1261	6102	**4.84**	4.56	5.14	< 0.0001
		94804	Richmond	1415	4756	**3.36**	3.17	3.57	< 0.0001
		94805	Richmond	271	764	**2.82**	2.46	3.23	< 0.0001
		94806	San Pablo	1743	5690	**3.26**	3.10	3.44	< 0.0001
	No	Not warned BAY (4–10 mi)		1819	2784	**1.53**	1.44	1.62	< 0.0001
		94530	El Cerrito	310	438	**1.41**	1.22	1.63	< 0.0001
		94803	El Sobrante	557	994	**1.78**	1.61	1.98	< 0.0001
		94564	Pinole	374	499	**1.33**	1.17	1.52	< 0.0001
		94547	Hercules	342	505	**1.48**	1.29	1.69	< 0.0001
		94572	Rodeo	236	398	**1.69**	1.44	1.98	< 0.0001
		Rest of County (10+ mi)		17988	18892	1.05	1.03	1.07	< 0.0001

**Fig. 3:**
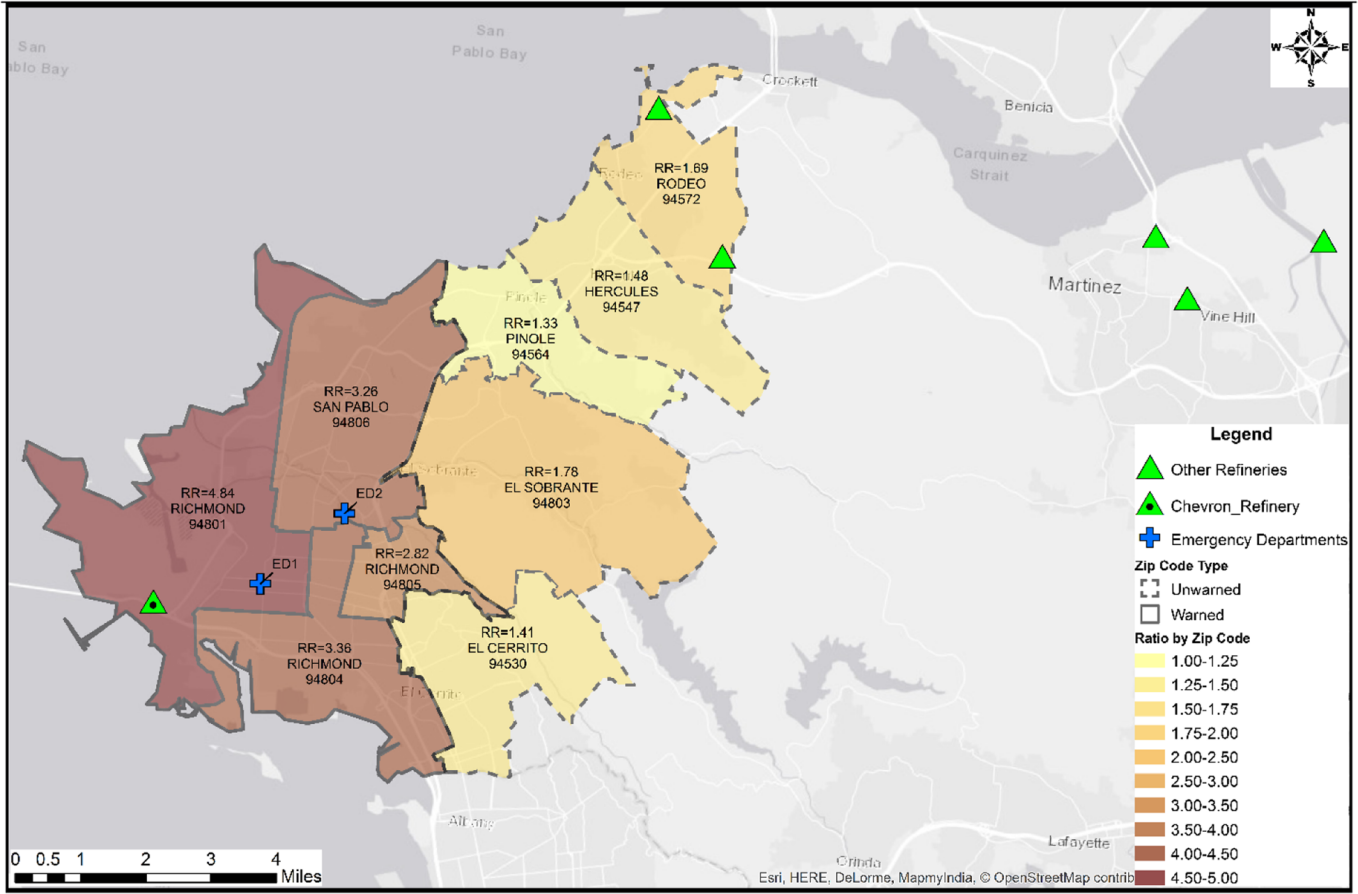
2012 CRI ratio increase in ED visits per 1 K population, by warned status

Figure 3 is a map of the affected areas, with the west and northwest border on San Francisco Bay, with ROC not shown to the east and northeast, and Alameda County to the south. Locations of the subject Refinery and the two EDs in this area are identified, as are other refineries in Rodeo and Martinez. The subject Refinery and other refineries have had CRI since 2012 [1]. In the 2007 and 2012 CRI, officials issued SIP warnings (solid boundaries) in Richmond (ZIP 94801 (includes North Richmond), 94804, 94805), and San Pablo (ZIP 94806) but did not warn other Bayside towns (dashed boundaries). The Hayward Fault upwelling is along the eastern boundaries of unwarned ZIPs. The map repeats the ZIP-level 2012 RR from Table 3.

**Fig. 4:**
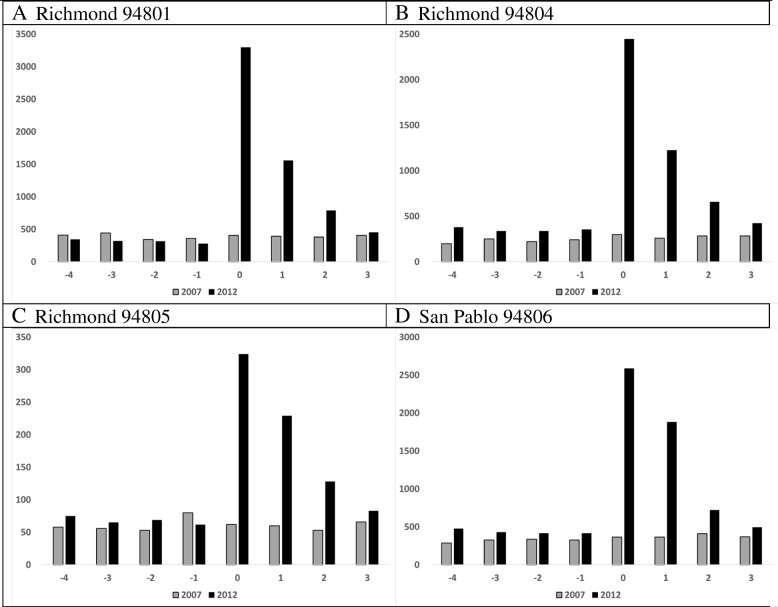
Communities warned to shelter in place, ED visits by week, 2007 and 2012

Figure 4 shows ED visits by residents of warned towns for the 4 weeks before and after the 2007 and 2012 CRI (Table 3, RR range. 2.8–4.8 in 2012). In Week 0, the absolute number of visits increased 7- to 10-fold and, depending on area, did not return to normal until Week 3.

**Fig. 5:**
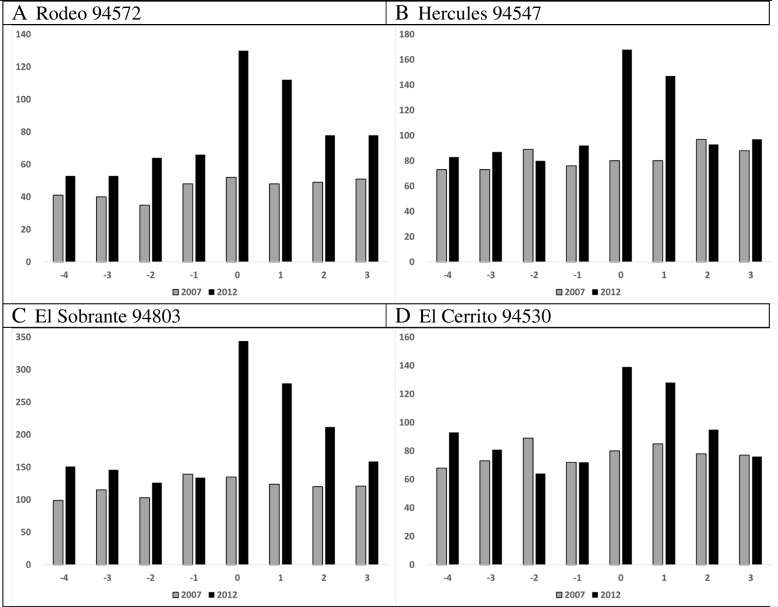
Communities not warned to shelter in place, ED visits by week, 2007 and 2012

Figure 5 turns to BAY communities not warned to SIP. In the 2012 CRI, ED visits increased significantly (Table 3, RR range 1.33–1.69), and number of weeks to return to pre-CRI numbers varied by town. To simplify the figure, we do not show Pinole.

## Discussion

Consistent with the review article highlighting the paucity of population-based quantitative studies [20], the only approximately comparable CRI report we found used California ED and PD data to focus on forest fires. Wettstein and colleagues [18] reported the expected increases in respiratory conditions but also identified that cardiovascular events were prominent in the wild fire exposed population. We did not find the increase in heart attacks that Wettstein reported. This may be due to the magnitude of stress experienced by people fleeing enormous wildfires with attendant loss of treasured personal belongings, pets and farm animals, homes, and even entire communities.

Over 2000 residents of communities warned to SIP during the 2012 CRI filed a lawsuit against the Refinery [50]. In 2017, the court ordered physical examinations of 40 randomly selected plaintiffs, with medical histories and medical records review. The court confined the physicals to assessing head, eyes, ears, nose, throat, lungs, heart, and deep tendon reflex, and limited the report to the number seeking urgent medical care, those reporting wheezing within several days, and those whose physicians reported worsening of existing asthma or introduction of more powerful asthma medications within 4 weeks. As confirmed by health interviews conducted 5 years after the 2012 CRI, most conditions were transient, frightening, but not life threatening. However, half (*N* = 20) had no respiratory symptoms before the 2012 event and of those, 5 remained symptomatic. Of 20 plaintiffs with respiratory conditions before 2012, symptoms returned to pre-event levels for 5 and were worse for 15. Thus, about half of plaintiffs had new or worsened chronic respiratory conditions. This is consistent with reports of children’s respiratory outcomes after the 1989 Loma Prieta earthquake that also affected this community [11].

The BAAQMD air monitoring network did not capture localized impacts from short duration incidents like the 2012 Refinery fire [33]. Meaningful monitoring requires routine analysis of dozens of compounds, more than the 23 that BAAQMD sampled after the 2012 event. No monitors in the Refinery’s vicinity sampled PM 2.5, the cause of acute health effects. In this short-term, high-intensity event emissions impacted areas without air monitors. An abundance of well-placed monitors is needed to provide an accurate picture of the impacts of CRI events such as a refinery fire. This did not exist in 2012. Both a denser permanent network in areas near the refineries and greater capacity for mobile short-term monitoring are advised.

With no functioning or properly placed monitors, no air quality reports exist to determine the levels or types of chemicals released. In such circumstances, alternative resources may help to understand the health impact. We turned to ED records to study changes in presenting diseases as an alternative. This approach will not identify chemicals released but will identify types of illness to expect from a similar CRI involving petroleum by-products. It may also help EDs plan their responses for future CRI.

In both the 2007 and 2012 event, the County issued SIP warnings to the same communities. Both times, the same ineffective monitoring systems were in place. The 2007 event caused a modest but significant increase in ED utilization that the 2012 event dwarfed, and both events increased rates in the same body systems (Table 2). From this we conclude that both CRI released similar toxics, with releases of different magnitudes.

Initial reports after the 2012 CRI ascribed the increase in ED utilization to the notion that residents were nervous because of the SIP. However, communities that did not receive SIP warnings in either 2007 or 2012 experienced statistically significant increases in ED utilization (Table 3). During the 2007 CRI, the four nearby ZIPs warned to SIP showed divergent results, with the two northerly ones showing significant increases (RR = 1.18 and 1.23), while the two southerly ones showed no increase (RR = 1.02 and 0.98). This is consistent with a more northerly direction of the 2007 CRI plume, and is inconsistent with the hypothesis that warning the public to SIP, by itself, caused an increase in ED visits.

From this we conclude that SIP warning did not cause the surge, but rather the unmeasured magnitude of the CRI events drove thousands of people to seek emergency care. This puts into focus the public health consequences of officials making decisions that are not data-driven [14]. It also highlights the family and community burden of such events in terms of losing work days, scrambling to find child care, and traveling long distances with sick family members to obtain care amidst the vagaries of Bay Area traffic.

In the context of the relatively mild uptick in ED utilization from the 2007 event, state, regional, county, and local officials did not require the Refinery (and other County refineries) to properly maintain and appropriately place monitoring equipment and to report routine chemical release data. As of the writing of this paper, we do not know if any government agency has reported the full extent of emissions from the 2012 Refinery incident [51]. Although BAAQMD recommended adding “mobile monitoring capabilities to better assess incidents in the future [52]”, we did not find any reports that they have done so.

The subject Refinery is one of a number ringing the County’s west and north borders (Fig. 3), and one of the more risky from the point of prevailing winds blowing from the shores of San Francisco Bay. Since the 2012 event, area refineries continue to have CRI [1], and a Rodeo refinery currently is seeking approval to become a major processor of tar sand oil in the United States [6, 7, 53]. We strongly recommend that the County review and consider expanding its SIP warning areas.

The enormous 2012 census increase in only two small EDs was caused both by the magnitude of the unmeasured emissions and also by the paucity of ED and general acute care facilities in this highly populated urban area. The BAY region has only 50 in-patient beds [54, 55]. From the Refinery, it is a 45-min drive to the next nearest County inpatient hospital, and a 30-min drive to the nearest Alameda County hospital, subject to conditions on the most heavily trafficked roads in Northern California.

In the 1970s, California passed comprehensive legislation to create a framework to control hospital costs yet protect patient safety. The legislation required an inventory of health facilities, planning processes including regional Health Service Areas, local Health Facilities Planning Areas, and Certificates of Need to justify closures or service expansions, development of data systems to monitor utilization, costs, and expenditures and safety-related policies to monitor hospital structures, processes of care, and patient care [56, 57].

In 1987, responding to intense hospital industry lobbying, the legislature suspended the Certificate of Need and related planning activities and permitted hospitals to close or consolidate without state review. Unlike states that kept Federal planning requirements in place, California deregulated its healthcare system, finding it “indispensable that providers of health care be free to engage in voluntary, cooperative efforts with consumers, government, or other providers of health care to fulfill the purposes of the health planning laws [58]”. In a series of studies, Remy and colleagues documented the consequences of deregulation for access to care for California’s children, pregnant women, and the mentally ill [59-61].

The County population grew 45% from 803732 in 1990 [62] to 1165190 in 2019 [63]. Between the 1987 deregulation and the 2012 CRI, one hospital in the County closed, three hospitals closed their ED, and the publicly-owned district hospital housing ED2 closed its inpatient facility with California’s only burn unit north of San Francisco. Two years after the 2012 CRI, ED2 closed. Today, one hospital with ED1 and 50 inpatient beds serves one-quarter of the County population in the BAY region. Bounded on the east by the upwelling of the Hayward Fault, the region is lower income and has poorer health due at least in part to ongoing refinery-derived air pollution [30, 31]. By contrast, 9 hospitals with 6 EDs and about 1800 inpatient beds serve about three-quarters of the County population living in higher-income, lower-risk communities. Given population growth and hospital changes, the regional health infrastructure will be increasingly challenged to respond to future CRI or other major disasters.

## Conclusions

It is hard to imagine conditions faced by medical staff in two small Emergency Departments typically treating about 80 people over a 24-h day, but they managed to provide urgent care to thousands of people following a major industrial accident in 2012. Court-ordered examinations of randomly-selected plaintiffs showed that many patients emerged with worsened chronic health conditions. The importance of adequate air monitoring and emergency warning systems is highlighted by their absence when these events occurred. Warnings did not cause people to seek care. CRI emissions caused people to seek care. In this context, the data strongly suggests that more areas need to receive SIP warnings. In 2014, Emergency Department capacity in the area was reduced by half. The County’s growing population combined with its decreased and unevenly distributed hospital capacity raises concerns about the ability to provide adequate care during future events of similar or worse magnitude.

## Additional files

Additional file 1: List of AHRQ/CCS diagnosis groupers. (PDF 31 kb)

Additional file 2: Short list of diagnosis groups within body system. (PDF 45 kb)

## Abbreviations

AHRQ: Agency for Healthcare Research and Quality
BAAQMD: Bay Area Air Quality Management District
CCS: Multi-Level Clinical Classification Software
CI: 95% confidence interval
CRI: Chemical release incident
DXCH: CCS Body-system level diagnosis classification
DXCL: CCS Diagnosis group class within body system
ED: Emergency Department
LCL: Lower confidence limit
PD: Patient discharge data
PDX: Principal diagnosis
RR: Relative risk or risk ratio
SIP: Shelter in place
UCL: Upper confidence limit
