# Supplementary material for: Hospital, health, and community burden after oil refinery fires, Richmond, California 2007 and 2012
Source: Environ Health. 2019 May 16;18:48. doi: 10.1186/s12940-019-0484-4 (PMC6524223; doi:10.1186/s12940-019-0484-4)
Supplement: Supplementary file 1 — List of AHRQ/CCS diagnosis groupers. (PDF 31 kb) [file 12940_2019_484_MOESM1_ESM.pdf]

Supplemental file 1: Diagnosis groupers

Grouper	Conditions
**01 Infectious and parasitic diseases**	01 Tuberculosis; 02 Septicemia (except in labor); 03 Bacterial infection, unspecified site; 04 Mycoses; 05 HIV infection; 06 Hepatitis; 07 Viral infection; 08 Other infections, including parasitic; 09 Sexually transmitted infections (not HIV hepatitis); 10 Immunization screening for infectious disease
**02 Neoplasms**	11 Head/neck; 12 Esophageal; 3 Stomach ; 14 Colon; 15 Rectum/anus; 16 Liver/intrahepatic bile duct; 17 Pancreas;18 Gastrointestinal/peritoneal;19 Bronchial/lung; 20 Other respiratory; 21 Bone/connective tissue; 22 Skin melanoma; 23 Non-epithelial; 24 Breast; 25 Uterus; 26 Cervix; 27 Ovary; 28 Female genital; 29 Prostate; 30 Testis; 31 Male genital; 32 Bladder; 33 Kidney/renal; 34 Urinary organ;35 Brain/ns can; 36 Thyroid; 37 Hodgkin’s lymphoma; 38 Non-Hodgkin’s lymphoma; 39 Leukemia; 40 Multiple myeloma; 41 Other primary cancer; 42 Secondary malignancy; 43 Malignant neoplasm; 44 Neoplasm unspecified; 45 Maintenance chemotherapy; 46 Benign uterine neoplasm; 47 Other benign neoplasm
**03 Endocrine; nutritional; and metabolic diseases and immunity disorders**	48 Thyroid disorders; 49 Diabetes mellitus without complication; 5Diabetes mellitus with complications; 51 Other endocrine disorders; 52 Nutritional deficiencies; 53 Disorders lipid metabolism; 54 Gout other crystal arthropathies; 55 Fluid electrolyte disorders; 56 Cystic fibrosis; 57 Immunity disorders; 58 Other nutritional, endocrine, metabolic disorders
**04 Diseases of the blood and blood-forming organ**	59 Anemia; 60 Acute post-hemorrhagic anemia; 61 Sickle cell anemia; 62 Coagulation and hemorrhagic disorders; 63 Diseases of white blood cells; 64 Other hematologic conditions
**05 Mental Illness**	650 Adjustment disorders; 651 Anxiety disorders; 652 Attention-deficit/conduct/disruptive behavior; 653 Delirium/dementia/amnestic/other cognitive; 654 Developmental disorders; 655 Disorders usually diagnosed in infancy/childhood; 656 Impulse control disorders NEC; 657 Mood disorders; 658 Personality disorders; 659 Schizophrenia and other psychotic disorders; 660 Alcohol-related disorders; 661 Substance-related disorders; 662 Suicide and intentional self-inflicted injury; 663 Screening and history of mental health; 670 Miscellaneous mental disorders; 653 Delirium/dementia/amnestic/other cognitive; 654 Developmental disorders; 660 Alcohol-related disorders; 663 Screening and history of mental health; 670 Miscellaneous mental disorders
**06 Diseases of the nervous system and sense organs**	76 Meningitis (exc caused by TB STD); 77 Encephalitis (except caused by TB STD); 78 Other CNS infection poliomyelitis; 79 Parkinson’s disease; 80 Multiple sclerosis; 81 Other hereditary degenerative nervous system conditions; 82 Paralysis; 83 Epilepsy, convulsions; 84 Headache, including migraine; 85 Coma, stupor, brain damage; 86 Cataract; 87 Retinal detachments, defects, vascular occlusion, retinopathy; 88 Glaucoma; 89 Blindness vision defects; 90 Inflammation, infection eye (except caused by TB STD); 91 Other eye disorders; 92 Otitis media related conditions; 93 Conditions associated with dizziness vertigo; 94 Other ear sense organ disorders; 95 Other nervous system disorders
**07 Diseases of the circulatory system**	96 Heart valve disorders; 97 Peri-, endo-, myocarditis, cardiomyopathy (except caused by TB STD); 98 Essential hypertension; 99 Hypertension with complications secondary hypertension; 100 Acute myocardial infarction; 101 Coronary atherosclerosis other heart disease; 0102 Nonspecific chest pain; 103 Pulmonary heart disease; 104 Other ill-defined heart disease; 105 Conduction disorders; 106 Cardiac dysrhythmias; 107 Cardiac arrest ventricular fibrillation; 108 Congestive heart failure, non-hypertensive; 109 Acute cerebrovascular disease; 110 Occlusion stenosis pre-cerebral arteries; 111 Other ill-defined cerebrovascular disease; 112 Transient cerebral ischemia; 113 Late effects cerebrovascular disease; 114 Peripheral visceral atherosclerosis; 115 Aortic, peripheral, visceral artery aneurysms; 116 Aortic peripheral arterial embolism thrombosis; 117 Other circulatory disease; 118 Phlebitis, thrombophlebitis thromboembolism; 119 Varicose veins lower extremity; 120 Hemorrhoids; 121 Other diseases veins lymphatics
**08 Diseases of the respiratory system;**	122 Pneumonia (exc caused by TB STD); 123 Influenza; 124 Acute chronic tonsillitis; 125 Acute bronchitis; 126 Other upper respiratory infections; 127 Chronic obstructive pulmonary disease bronchiectasis; 128 Asthma; 129 Aspiration pneumonitis, food/vomitus; 130 Pleurisy, pneumothorax, pulmonary collapse; 131 Respiratory failure, insufficiency, arrest (adult); 132 Lung disease due to external agents; 133 Other lower respiratory disease; 134 Other upper respiratory disease
**09 Diseases of the digestive system**	135 Intestinal infection; 136 Disorders teeth jaw; 137 Diseases mouth, excluding dental; 138 Esophageal disorders; 139 Gastroduodenal ulcer (except hemorrhage); 140 Gastritis duodenitis; 141 Other disorders stomach duodenum; 142 Appendicitis other appendiceal conditions; 143 Abdominal hernia; 144 Regional enteritis ulcerative colitis; 145 Intestinal obstruction without hernia; 146 Diverticulosis diverticulitis; 147 Anal rectal conditions; 148 Peritonitis intestinal abscess; 149 Biliary tract disease; 151 Other liver diseases; 152 Pancreatic disorders (not diabetes); 153 Gastrointestinal hemorrhage; 154 Noninfectious gastroenteritis; 155 Other gastrointestinal disorders

**10 Diseases of the genitourinary system**	156 Nephritis, nephrosis, renal sclerosis; 157 Acute unspecified renal failure; 158 Chronic renal failure; 159 Urinary tract infections; 160 Calculus urinary tract; 161 Other diseases kidney ureters; 162 Other diseases bladder urethra; 163 Genitourinary symptoms ill-defined conditions; 164 Hyperplasia prostate; 165 Inflammatory conditions male genital organs; 166 Other male genital disorders; 167 Nonmalignant breast conditions; 168 Inflammatory diseases female pelvic organs; 169 Endometriosis; 170 Prolapse female genital organs; 171 Menstrual disorders; 172 Ovarian cyst; 173 Menopausal disorders; 174 Female infertility; 175 Other female genital disorders
**11 Complications of pregnancy; childbirth; and the puerperium**	176 Contraceptives; 177 Spontaneous abortion; 178 Induced abortion; 179 Abortion complications; 180 Ectopic pregnancy; 181 Other pregnancy complications; 182 Hemorrhage during pregnancy; 183 Hypertension in pregnancy; 184 Early labor; 185 Long pregnancy; 186 Diabetes mellitus in pregnancy; 187 Malposition; 188 Pelvic obstruction; 189 Previous Cesarean section; 190 Fetal distress; 191 Amniotic cavity; 192 Umbilical cord; 193 OB-related perineal trauma; 194 Forceps delivery; 195 Other complications of birth; 196 Normal pregnancy/delivery
**12 Diseases of skin and subcutaneous tissue**	197 Skin infection; 198 Other inflammation of the skin; 199 Ulcer skin; 200 Other skin diagnosis
**13 Diseases of the musculoskeletal system and connective tissue**	201 Infectious arthritis; 202 Rheumatic arthritis ; 203 Osteoarthritis ; 204 Other joint diagnosis ; 205 Back problem; 206 Osteoporosis; 207 Pathologic fracture; 208 Acquired foot defect; 209 Other acquired deformity; 0210 SLE; 211 Other connective tissue; 212 Other bone diagnosis
**14 Congenital anomalies**	213 Cardiac; 214 Gastrointestinal; 215 Genitourinary; 216 Nervous system; 217 Other congenital anomalies
**15 Certain conditions originating in the perinatal period**	218 Liveborn; 219 Low birth weight; 220 Birth asphyxia; 221 Respiratory distress; 222 Perinatal jaundice; 223 Birth trauma; 224 Other perinatal diagnosis
**16 Injury and poisoning**	0225 Joint injury; 226 Hip fracture; 227 Spinal cord injury; 228 Face/Skull fracture; 229 Arm fracture; 230 Leg fracture; 231 Other fracture; 232 Sprain; 233 Intracranial injury; 234 Crush injury; 235 Open wound head; 236 Open wound extremity; 237 Complication of device ; 238 Complication of procedure; 239 Superficial injury; 240 Burns; 241 Poison psychotropic; 242 Poison other medicine; 243 Poison non-medicine; 244 Other injury
**17 Symptoms; signs; and ill-defined conditions and factors influencing health status**	245 Syncope; 246 FUO; 247 Lymph enlargement; 248 Gangrene; 249 Shock; 250 Nausea/vomiting; 251 Abdominal pain; 252 Fatigue; 253 Allergy; 254 Rehabilitation; 255 Social adminstration; 256 Exam/evaluate; 257 Other aftercare; 258 Other screen; 259 Unclassified
**18 Residual codes; all E codes**	2601 E Codes: Cut/pierce; 2602 E Codes: Drowning/submersion; 2603 E Codes: Fall; 2604 E Codes: Fire/burn; 2605 E Codes: Firearm; 2606 E Codes: Machinery; 2607 e codes: motor vehicle traffic (mvt); 2608 E Codes: Pedal cyclist- not MVT; 2609 E Codes: Pedestrian- not MVT; 2610 e codes: transport- not mvt; 2611 E Codes: Natural/environment; 2612 E Codes: Overexertion; 2613 E Codes: Poisoning; 2614 E Codes: Struck by- against; 2615 E Codes: Suffocation; 2616 E Codes: Adverse effects of medical care; 2617 E Codes: Adverse effects of medical drugs; 2618 E Codes: Other specified and classifiable; 2619 e codes: other specified- nec; 2620 E Codes: Unspecified; 2621 E Codes: Place of occurrence
**Other**	SAS code available on request for all constructed variables.
