# Supplementary material for: Hospital, health, and community burden after oil refinery fires, Richmond, California 2007 and 2012
Source: Environ Health. 2019 May 16;18:48. doi: 10.1186/s12940-019-0484-4 (PMC6524223; doi:10.1186/s12940-019-0484-4)
Supplement: Supplementary file 2 — Short list of diagnosis groups within body system. (PDF 45 kb) [file 12940_2019_484_MOESM2_ESM.pdf]

Body System	Diagnosis group	Principal Diagnosis (N) Before	After	Total	Patient Percent Before	After	Difference Abs	Pct	Rel Risk
06 Nervous system/sens	0084 Headache/mig	656	2,828	3,484	2.7	7.2	4.6	81.2	4.31
	0090 Eye infectn	182	1,153	1,335	0.7	3.0	2.2	86.4	6.34
	0091 Other eye dx	105	1,170	1,275	0.4	3.0	2.6	91.8	11.14
	0093 Dizziness	283	481	764	1.2	1.2	0.1	63.0	1.70
	0095 Oth nerv dx	339	410	749	1.4	1.1	(0.3)	54.7	1.21
07 Circulatory system	0102 Chest pain	913	1,372	2,285	3.7	3.5	(0.2)	60.0	1.50
08 Respiratory system	0126 Ot up rsp in	620	1,332	1,952	2.5	3.4	0.9	68.2	2.15
	0128 Asthma	279	806	1,085	1.1	2.1	0.9	74.3	2.89
	0133 Oth low resp	452	2,047	2,499	1.8	5.2	3.4	81.9	4.53
	0134 Ot uppr resp	172	1,171	1,343	0.7	3.0	2.3	87.2	6.81
16 Injury/poisoning	0243 Poisn nonmed	59	3,065	3,124	0.2	7.9	7.6	98.1	51.95
	0244 Other injury	843	1,112	1,955	3.4	2.8	(0.6)	56.9	1.32
17 Symptoms; signs; ill-defined conditions and factors influencing health status	0246 FUO	218	462	680	1.1	2.1	1.0	67.9	2.12
	0250 Nausea/vomit	361	648	1,009	1.5	1.7	0.2	64.2	1.80
	0255 Social admin	383	1,020	1,403	1.6	2.6	1.0	72.7	2.66
	0259 Unclassified	258	530	788	1.1	1.4	0.3	67.3	2.05
